# Impact of genetic variants of *ABCB1, APOB, CAV1,* and *NAMPT* on susceptibility to pancreatic ductal adenocarcinoma in Chinese patients

**DOI:** 10.1002/mgg3.1226

**Published:** 2020-04-03

**Authors:** Baohuan Li, Chuanzhen Zhang, Jingjing Wang, Meijuan Zhang, Changhong Liu, Ziping Chen

**Affiliations:** ^1^ Department of Gastroenterology Shandong Provincial Qianfoshan Hospital Shandong University Jinan China; ^2^ Department of Gastroenterology The First Affiliated Hospital of Shandong First Medical University Shandong Provincial Qianfoshan Hospital Jinan China; ^3^ Clinical Laboratory The First Affiliated Hospital of Shandong First Medical University Shandong Provincial Qianfoshan Hospital Jinan China

**Keywords:** *ABCB1*, *APOB*, *CAV1*, genetic polymorphism, *NAMPT*, pancreatic ductal adenocarcinoma

## Abstract

**Background:**

Among the different types of cancer, pancreatic cancer, particularly pancreatic ductal adenocarcinoma (PDAC), is the most lethal malignancy, with poor early detection rates and prognosis. The aim of the present study was to investigate the potential genetic effects of the single‐nucleotide polymorphisms (SNPs) in *ABCB1* (rs1045642, rs3789243, rs4148737), *APOB* (rs693, rs1042031), *CAV1* (rs12672038, rs1997623, rs3807987, rs7804372), and *NAMPT* (rs9034, rs2505568, rs61330082) on PDAC.

**Methods:**

A total of 273 patients with PDAC and 263 healthy controls were genotyped using PCR and direct Sanger sequencing. Unconditional logistic regression models were used to evaluate the potential effects of the genotypes, alleles, and haplotypes on the risk of developing PDAC.

**Results:**

Patients with PDAC possessed a considerably lower frequency of genotypes AG, GG, and allele G at *ABCB1* rs4148737 compared with controls. Based on age, sex, smoking status, drinking status, diabetes, and family history of cancer, stratified analyses showed a significant correlation between SNPs at rs4148737 and PDAC. According to specific SNPs, eight haplotypes were constructed along with *ABCB1* rs4148737, rs1045642, and rs3789243. Carriers with haplotypes ACC and ATC were more susceptible to developing PDAC, whereas haplotypes GCC and GTC were associated with a reduced likelihood of developing PDAC. The distributions of the other SNPs in each group were not significantly associated with PDAC risk.

**Conclusions:**

These results suggested that genetic polymorphisms of *ABCB1* rs4148737 may influence an individual's risk of developing PDAC.

## Graphical abstract

Pancreatic ductal adenocarcinoma is the most lethal cancer with lower morbidity. We selected several potential SNPs, administrated a case‐control study in China by examining their genotypes, constructing haplotypes, and evaluating the effects of positive SNP by stratification analysis. Interestingly, we found that *ABCB1* rs4148737 might influence the genetic susceptibility of PDAC.

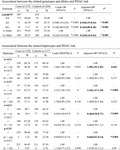

## 1 INTRODUCTION

Pancreatic cancer is one of the hardest to treat malignancies. According to GLOBOCAN 2018 estimates, pancreatic cancer is the 11th most common type of cancer worldwide, with 458,918 new cases (accounting for 2.5% of all cases of cancer) and 432,242 deaths (4.5% of all deaths caused by cancer) (Bray et al., 2018). Additionally, the number of cancer death ranked the seventh in developed countries (Bray et al., 2018), and its number of cancer death ranked third in the USA (Siegel, Miller, & Jemal, 2019). In China, it was the 10th most common malignant carcinoma in 2015 (2.42% of all cancer cases), and it is the sixth leading cause of cancer‐associated death (3.64% of all cancer‐associated deaths) (Zheng et al., 2019).

The etiology of pancreatic cancer has been extensively studied by numerous meta‐analyses and pooled analyses. Thus far, several risk factors can be divided into two primary categories: Modifiable and non‐modifiable. The former includes smoking, alcohol, dietary factors, obesity, and exposure to toxic substances, whereas the latter includes ethnicity, sex, age, family history of pancreatic cancer, diabetes mellitus, genetic factors, chronic infections, non‐O blood group, and chronic pancreatitis (Midha, Chawla, & Garg, 2016). However, the specific etiology has been not defined. Although several pathological types of pancreatic malignancies have been identified, pancreatic ductal adenocarcinoma (PDAC) and pancreatic neuroendocrine tumor are the most prevalent, which account for 90% and 5%, respectively (Hackeng, Hruban, Offerhaus, & Brosens, 2016). Additionally, patients with an early stage of pancreatic cancer do not typically exhibit symptoms. However, as the cancer progresses, it may physically manifest as a gradual onset of nonspecific symptoms, such as jaundice, light‐colored stools, abdominal pain, weight loss, and fatigue (Siegel, Miller, & Jemal, 2018). The available diagnostic tests for detection include measuring the blood levels of cancer antigen 19–9, abdominal ultrasonography, tri‐phasic pancreatic protocol CT, magnetic resonance imaging, and endoscopic ultrasound‐guided fine‐needle aspiration for cytological diagnosis (the sensitivity of which is reported to be ~80%) (Longnecker, Karagas, Tosteson, & Mott, 2000). Nonetheless, they are nonspecific and may miss numerous patients with early stage pancreatic cancer (De La Cruz, Young, & Ruffin, 2014). Therefore, pancreatic cancer is usually diagnosed at the advanced stage, and 80%–90% of patients present with unresectable tumors at the first diagnosis (Rawla, Sunkara, & Gaduputi, 2019). Once a definite diagnosis has been made, surgery, chemotherapy, and radiotherapy are traditionally used to extend the patients’ survival and/or relieve their symptoms. However, for patients with advanced‐stage pancreatic cancer, there is still no definitive or effective cure (Mohammed, Van Buren, & Fisher, 2014).

Prevention, early detection, and curing the disease in the early stages are key to reduce the morbidity and mortality rates. Novel etiological agent or more specific diagnostic tools may result in improved outcomes for patients. Thus, there is a need for ongoing evaluation for the epidemiology of this malignancy. Among the factors which need to be assessed, genetic variations or mutations serve an important role in the increased risk of pancreatic cancer (Ghiorzo, 2014). Of the patients with pancreatic cancer, ~10% possess a genetic predisposition to developing the malignancy (Shi, Daniels, & Hruban, 2008). Several germline mutations have been demonstrated to be involved in hereditary forms of pancreatic cancer, such as *BRCA1* (OMIM # 113,705)*, BRCA2* (OMIM # 600,185)*, PALB2* (OMIM # 610,355)*, ATM* (OMIM # 607,585)*, APC* (OMIM # 611,731)*, MLH1* (OMIM #120436)*, MSH2* (OMIM # 609,309)*, MSH6* (OMIM # 600,678)*, PMS2* (OMIM # 600,259)*, PRSS1* (OMIM # 276,000), and *STK11* (OMIM # 602,216) (Solomon, Das, Brand, & Whitcomb, 2012; Vincent, Herman, Schulick, Hruban, & Goggins, 2011).

The ATP‐binding cassette subfamily B member 1 (*ABCB1* [OMIM #171050]), also known as multidrug resistance gene 1, encodes a transmembrane P‐glycoprotein (P‐gp). This protein functions to efflux of endogenous metabolites and toxic xenobiotics, including intracellular carcinogens, suggesting its protective role against carcinogenesis. P‐gp also serves an important role in the reduction of drug response, through modulating absorption, metabolism, and promoting elimination of drugs from cells. P‐gp also activates lymphocytes during the immune response (Hodges et al., 2011). It is speculated that genetic polymorphisms of *ABCB1* may result in variations in the expression levels of the mRNA transcript and protein, or result in misfolded proteins, and reduced or altered substrate specificity, which will likely influence drug pharmacokinetics or disease outcome (Fung & Gottesman, 2009). Several studies have reported the association between *ABCB1* polymorphisms with cancer risk, and the results were not consistent absolutely (He, Mo, Zhang, & Liu, 2013; Ruiz‐Pinto et al., 2016; Sabahi et al., 2010; Sam et al., 2007; Yan et al., 2019).

Obesity is associated with an increased risk of pancreatic cancer (Davoodi, Malek‐Shahabi, Malekshahi‐Moghadam, Shahbazi, & Esmaeili, 2013) and it may increase the incidence and mortality of pancreatic cancer (Berrington de Gonzalez, Sweetland, & Spencer, 2003; Calle, Rodriguez, Walker‐Thurmond, & Thun, 2003). Abnormal lipid metabolism promotes pancreatic tumorigenesis (Guillaumond et al., 2015; Ying et al., 2016). The apolipoprotein B (APOB) protein forms an integral part of chylomicrons and very low‐density lipoproteins during lipoprotein metabolism. Several SNPs in *APOB* (OMIM # 107,730) have been reported, although their significance to cancer has not been studied, to the best of our knowledge. Therefore, whether they influence the progression of pancreatic carcinoma remains to be determined.

Caveolin 1 (CAV1) serves an important role in cell signaling regulation and acts as a scaffold involved in various signaling ways. For example, the caveolin‐scaffolding domain negatively regulates the activity of numerous signaling molecules, including endothelial nitric oxide synthase (Ju, Zou, Venema, & Venema, 1997) and epidermal growth factor receptor (Couet, Sargiacomo, & Lisanti, 1997). *CAV1* (OMIM # 601,047) is expressed in a variety of tumor cells and exhibits carcinogenic and tumor suppressive functions dependent on the specific type of cancer and stage (Gupta, Toufaily, & Annabi, 2014). Taken together, previous studies suggested that *CAV1* expression may inhibit tumor growth in the early stages of cancer, and promote its invasion and metastasis in the later stages (Shatz & Liscovitch, 2008). To date, numerous studies have explored the association between genetic polymorphisms of *CAV1* and hepatocellular, colorectal, and gastric cancers (Hsu et al., 2013; Yang et al., 2010; Zhang, Hu, et al., 2014).

Nicotinamide phosphoribosyltransferase (NAMPT) was initially identified as a pre‐B‐cell colony‐enhancing factor. NAMPT is also a rate‐limiting enzyme in NAD biosynthesis, which is crucial for numerous vital cellular processes, genomic stability, and organismal metabolic homeostasis (Shackelford, Mayhall, Maxwell, Kandil, & Coppola, 2013; Ying, 2008). Additionally, NAMPT is involved in angiogenesis by activating the extracellular signal‐regulated kinase 1/2 pathway, and increasing the expression of vascular endothelial growth factor and matrix metalloproteinase 2/9 (Kim et al., 2007). *NAMPT* (OMIM # 608,764) is highly evolutionarily conserved, suggesting that even small genetic variations may profoundly affect its protein expression, function, and subsequent dependent events.

Based on the above, certain SNPs of these proteins, which may affect protein expression, structure, or function, may be associated with genetic susceptibility to PDAC. However, to the best of our knowledge, there are no studies which have investigated the potential association between these SNPs and the risk of PDAC. Thus, the aim of the present study was to analyze the relationships between SNPs of *ABCB1* (rs1045642 [HGVS Name: g.208920T>C], rs3789243 [HGVS Name: g.126679T>C], rs4148737 [HGVS Name: g.176413A>G]), *APOB* (rs693 [HGVS Name: g.39751C>T], rs1042031 [HGVS Name: g.46193G>A]), *CAV‐1* (rs12672038 [HGVS Name: g.27268G>A], rs1997623 [HGVS Name: g.5522A>C], rs3807987 [HGVS Name: g.19996G>A], rs7804372 [HGVS Name: g.34390T>A]), and *NAMPT* (rs9034 [HGVS Name: c.*1473T>C], rs2505568 [HGVS Name: g.1929A>T], rs61330082 [HGVS Name: g.106286419G>A]), and the hereditary susceptibility of PDAC in Chinese patients.

## 2 MATERIALS AND METHODS

### 2.1 Study participants

Between January 2015 and December 2018, 273 patients with PDAC (165 males and 108 females) who visited Shandong Provincial Qianfoshan Hospital were recruited. All diagnoses were confirmed by qualified pathological analysis of surgical specimens or endoscopic ultrasound‐guided fine‐needle aspiration. Every slide was confirmed by two pathologists in Shandong Provincial Qianfoshan Hospital independently, while the disagreement was resolved by the senior pathologists. None of the patients recruited received chemotherapy or any other treatments for PDAC prior to recruitment. Moreover, patients with incomplete clinical information and histories of previous cancers or autoimmune diseases were excluded in this study.

The control group composed of 269 healthy and genetically unrelated individuals (130 males and 139 females), with age between 25 and 80 years, randomly selected from Shandong permanent residents over the same period. They were voluntary participants without any known history of caner, psychological disorders, autoimmune diseases of the pancreas, cardiocerebral vascular diseases, or infection with hepatitis B virus (HBV), hepatitis C virus (HCV), or human immunodeficiency virus (HIV).

Each participant provided written informed consent for participation in the study, and their demographic and lifestyle characteristics were collected and recorded by one‐on‐one interviews. A history of regular cigarette smoking was defined as ≥1 cigarette per day for ≥12 months, or ≥18 packs for 1 year, while individuals drinking Chinese liquor ≥50 ml, or beer ≥200 ml biweekly for 12 months were thought as positive history of drinking.

### 2.2 DNA extraction

A total of 3 ml of peripheral blood was drawn from each patient, and the genomic DNA was extracted using a Blood DNA kit according to the manufacturer's protocol (Omega Bio‐Tek, Inc.).

### 2.3 Genotyping

Genomic DNA was amplified using PCR. The sequencing primers used were the same as previous studies (Al‐Bustan, Alnaqeeb, Annice, Ebrahim, & Refai, 2014; Caronia et al., 2011; He et al., 2013; Wang, Zhang, Liu, Xu, & Chen, 2014; Zhang et al., 2015). PCR amplification was performed in a total of 25‐µl reaction mixture, containing 100–150 ng genomic DNA, 0.5 μl of each primer (10 μM each; Shanghai Boshang Biotechnology Co., Ltd.), 0.5‐μl dNTP (10 mM), and 1.5 U Taq DNA polymerase (Takara Bio, Inc.). The thermocycling conditions were: Initial denaturation for 2 min at 94°C; followed by 38 cycles of 30 s at 94.0°C, 50 s at the respective annealing temperatures and 1 min at 72.0°C; and a final extension step of 5 min at 72.0°C. All PCR products were run on 2% agarose gels and imaged. Genotypes were identified using direct Sanger sequencing (Shanghai Boshang Biotechnology Co., Ltd.).

### 2.4 Statistical analysis

Comparison of age distribution between the two groups was performed using a Mann–Whitney U test. Differences in the other demographic variables were compared using a McNemar test. To verify that all subjects were selected from a representative population, a χ^2^ goodness‐of‐fit test was used to test genotypes for Hardy–Weinberg equilibrium. PHASE software (version 2.1, University of Chicago) haplotype and haplotype frequencies were constructed and estimated. The odds ratios (ORs) and 95% confidence intervals (95% CIs) were calculated using unconditional logistic regression models. The adjusted ORs and 95% CIs were derived from logistic models adjusted for age, sex, smoking, alcohol consumption, diabetes, and family history of cancer. All statistical analyses were two‐sided and performed in Stata 15.1 (StataCorp LP). *p* < .05 was considered to indicate a statistically significant difference.

## 3 RESULTS

### 3.1 Distribution of patient characteristics

Table 1 shows the distribution of characteristics in the patients with PDAC and the healthy controls. The mean age (mean ± *SD*) of cases and controls were 64.56 + 12.11 years and 63.31 + 9.40 years, respectively, and there was no significant difference (*p* = .060). Alcohol consumption and incidence of diabetes were also not significantly different between the two groups (*p* = .086 and *p* = .093, respectively). There was a significant difference in the distribution of sex, tobacco consumption, and family history of cancer between the two groups (*p* = .005, *p* < .001, and *p* = .009, respectively). Specially, the frequencies of males, smokers, or individuals with family history of cancer were much higher in cases than controls (60.44% versus 48.33%; 30.77% versus 17.73%; 21.25% versus 12.64%, respectively).

**TABLE 1:** Distribution of genetic characteristics in cases and controls

Characteristics	Cases (*n* = 273)		Controls (*n* = 269)		*p*
	*n*	%	*n*	%	
Age (year) mean ± *SD*	64.56 ± 12.11		63.31 ± 9.40		.060^a^
Gender					
Male	165	60.44	130	48.33	.005^b^
Female	108	39.56	139	51.67	
Smoking					
Ever	84	30.77	45	16.73	<.001^b^
Never	189	69.23	224	83.27	
Drinking					
Ever	49	17.95	34	12.64	.086^b^
Never	224	82.05	235	87.36	
Diabetes					
Yes	51	18.68	36	13.38	.093^b^
No	222	81.32	233	86.62	
Family history of cancer					
Yes	58	21.25	34	12.64	.009^b^
No	215	78.75	235	87.36	

^a^ Mann–Whitney U test.

^b^ McNemar test.

### 3.2 Association between genetic polymorphisms and PDAC susceptibility

All genotype and allele frequencies of the SNPs examined are presented in Table 2. The distribution of these genotypes in each group did not deviate from the Hardy–Weinberg equilibrium (*p* > .05). After adjustment for confounding factors (age, sex, smoking, drinking, diabetes, and family history of cancer) in an unconditional logistic regression model, patients with PDAC possessed significantly lower frequencies of genotypes AG, GG, and allele G at rs4148737 compared with the controls (26.79% versus 45.73%, *p* < .001; 7.17% versus 21.79%, *p* < .001; 20.57% versus 44.66%, *p* < .001, respectively). Furthermore, subjects with genotypes AG, GG, and allele G were less susceptible to developing PDAC (OR = 0.29, 95% CI: 0.19–0.43; OR = 0.16, 95% CI: 0.09–0.30; OR = 0.32, 95% CI: 0.24–0.43, respectively). No significant differences were observed between the frequencies of any other SNPs between patients with PDAC and the control group (*p* > .05).

**TABLE 2:** Association between the related genotypes and alleles and PDAC risk

Genotype	Cases (*n* = 273)		Controls (*n* = 269)		Crude OR (95% CI)	*p*	Adjusted OR^a^ (95% CI)	*p* ^a^
	*n*	%	*n*	%				
rs1045642	267		238					
CC	82	30.71	72	30.25	1.00	—	1.00	—
CT	118	44.19	127	53.36	0.82 (0.54–1.22)	.323	0.76 (0.50–1.15)	.190
TT	67	25.09	39	16.39	1.51 (0.91–2.50)	.111	1.50 (0.89–2.51)	.126
C Allele	282	52.81	271	56.93	1.00	—	1.00	—
T Allele	252	47.19	205	43.07	1.18 (0.92–1.51)	.189	1.16 (0.90–1.49)	.256
rs3789243	265		244					
CC	115	43.4	111	45.49	1.00	—	1.00	—
CT	119	44.91	110	45.08	1.04 (0.72–1.51)	.818	1.04 (0.72–1.52)	.829
TT	31	11.7	23	9.43	1.30 (0.71–2.37)	.389	1.37 (0.74–2.52)	.315
C Allele	349	59.45	332	60.14	1.00	—	1.00	—
T Allele	238	40.55	220	39.86	1.10 (0.85–1.43)	.46	1.13 (0.87–1.48)	.355
rs4148737	265		234					
AA	175	66.04	76	32.48	1.00	—	1.00	—
AG	71	26.79	107	45.73	0.29 (0.19–0.43)	<.001	**0.29 (0.19–0.43)**	**<.001**
GG	19	7.17	51	21.79	0.16 (0.09–0.29)	<.001	**0.16 (0.09–0.30)**	**<.001**
A Allele	421	79.43	259	55.34	1.00	—	1.00	—
G Allele	109	20.57	209	44.66	0.32 (0.24–0.42)	<.001	**0.32 (0.24–0.43)**	**<.001**
rs693	262		228					
CC	234	89.31	199	87.28	1.00	—	1.00	—
TC	26	9.92	19	8.33	1.16 (0.63–2.17)	.632	1.15 (0.61–2.17)	.671
TT	2	0.76	10	4.39	0.17 (0.04–0.79)	.023	0.18 (0.04–0.85)	.030
C Allele	494	94.27	417	91.45	1.00	—	1.00	—
T Allele	30	5.73	39	8.55	0.65 (0.40–1.06)	.086	0.64 (0.39–1.06)	.083
rs1042031	267		243					
GG	231	86.52	211	86.83	1.00	—	1.00	—
AG	36	13.48	30	12.35	1.10 (0.65–1.84)	.729	1.04 (0.61–1.76)	.897
AA	0	0	2	0.82	—	—	—	—
G Allele	498	93.26	452	93	1.00	—	1.00	—
A Allele	36	6.74	34	7	0.96 (0.59–1.56)	.873	0.92 (0.56–1.50)	.728
rs12672038	267		242					
GG	146	54.68	134	55.37	1.00	—	1.00	—
AG	106	39.7	92	38.02	1.06 (0.73–1.52)	.764	1.05 (0.72–1.52)	.811
AA	15	5.62	16	6.61	0.86 (0.41–1.81)	.692	0.90 (0.43–1.92)	.793
G Allele	398	74.53	360	74.38	1.00	—	1.00	—
A Allele	136	25.47	124	25.62	0.99 (0.75–1.32)	.956	0.99 (0.75–1.32)	.969
rs1997623	267		245					
CC	248	92.88	219	89.39	1.00	—	1.00	—
AC	17	6.37	24	9.8	0.63 (0.33–1.20)	.155	0.66 (0.34–1.27)	.209
AA	2	0.75	2	0.82	0.88 (0.12–6.32)	.901	1.11 (0.15–8.07)	.919
C Allele	513	96.07	462	94.29	1.00	—	1.00	—
A Allele	21	3.93	28	5.71	0.68 (0.38–1.21)	.185	0.72 (0.40–1.29)	.969
rs3807987	260		232					
GG	158	60.77	142	61.21	1.00	—	1.00	—
AG	85	32.69	80	34.48	0.95 (0.65–1.40)	.812	0.93 (0.63–1.37)	.701
AA	17	6.54	10	4.31	1.53 (0.68–3.45)	.307	1.61 (0.71–3.67)	.256
G Allele	401	81.84	364	78.45	1.00	—	1.00	—
A Allele	89	18.16	100	21.55	1.08 (0.80–1.46)	.616	1.07 (0.79–1.45)	.669
rs7804372	267		241					
TT	158	59.18	144	59.75	1.00	—	1.00	—
AT	109	40.82	97	40.25	1.02 (0.72–1.46)	.895	1.00 (0.70–1.44)	.997
AA	0	0	0	0	—	—	—	—
T Allele	425	79.59	385	79.88	1.00	—	1.00	—
A Allele	109	20.41	97	20.12	1.02 (0.75–1.38)	.909	1.00 (0.73–1.37)	.997
rs9034	266		243					
CC	226	84.96	211	86.83	1.00	—	1.00	—
TC	40	15.04	26	10.7	1.44 (0.85–2.44)	.179	1.38 (0.81–2.36)	.242
TT	0	0	26	2.47	—	—	—	—
C Allele	492	92.48	448	85.17	1.00	—	1.00	—
T Allele	40	7.52	78	14.83	0.96 (0.60–1.52)	.857	0.96 (0.60–1.53)	.853
rs2505568	264		231					
TT	41	15.53	42	18.18	1.00	—	1.00	—
AT	223	84.47	189	81.82	1.21 (0.75–1.94)	.431	1.14 (0.71–1.85)	.580
AA	0	0	0	0	—	—	—	—
T Allele	305	57.77	273	59.09	1.00	—	1.00	—
A Allele	223	42.23	189	40.91	1.06 (0.82–1.36)	.673	1.04 (0.80–1.34)	.783
rs61330082	269		260					
CC	98	36.43	81	31.15	1.00	—	1.00	—
CT	91	33.83	94	36.15	0.80 (0.53–1.21)	.289	0.76 (0.50–1.16)	.201
TT	80	29.74	85	32.69	0.78 (0.51–1.19)	.246	0.73 (0.47–1.12)	.150
C Allele	287	53.35	256	49.23	1.00	—	1.00	—
T Allele	251	46.65	264	50.77	0.85 (0.67–1.08)	.181	0.83 (0.65–1.06)	.135

Bold values in this table indicates the significant differences (*P* < .05).

^a^ Unconditional logistic regression adjusted for risk factors (age, gender, tobacco smoking, alcohol consumption, diabetes, and family history of cancer).

### 3.3 Stratification analyses of ABCB1 genetic polymorphisms and PDAC susceptibility

To evaluate the effect of genetic polymorphism at rs4148737 on the susceptibility of PDAC, subjects were stratified based on age, sex, smoking status, drinking status, presence of diabetes, and family history of cancer (Table 3). Compared with genotype AA, significantly decreased frequencies of genotypes AG and GG were detected among male patients with PDAC (adjusted OR = 0.22, 95% CI: 0.13–0.37), female cases (adjusted OR = 0.27, 95% CI: 0.15–0.47), patients in different age brackets (<60‐year‐old, adjusted OR = 0.18, 95% CI: 0.09–0.36; 60‐ to 80‐year‐old, adjusted OR = 0.30, 95% CI: 0.19–0.49; >80‐year‐old, adjusted OR = 0.09, 95% CI: 0.01–0.69, respectively), patients who had previously smoked or did smoke (adjusted OR = 0.22, 95% CI: 0.10–0.51), patients who never smoked (adjusted OR = 0.25, 95% CI: 0.16–0.39), patients who consumed alcohol (adjusted OR = 0.21, 95% CI: 0.08–0.56), patients who did consume alcohol (adjusted OR = 0.25, 95% CI: 0.17–0.38), patients with diabetes (adjusted OR = 0.36, 95% CI: 0.13–0.99), patients without diabetes (adjusted OR = 0.24, 95% CI: 0.16–0.36), patients with a family history of cancer (adjusted OR = 0.11, 95% CI: 0.04–0.33), and without a family history (adjusted OR = 0.28, 95% CI: 0.19–0.42).

**TABLE 3:** Stratification analyses of rs4148737 genotypes and PDAC risk

Variable	rs4148737 (case/control)		*p* ^a^	Adjusted OR^a^ (95% CI)
	AA	AG + GG	AA	AG + GG
Gender				
Male	108/37	53/80	1.00	<0.001
				0.22 (0.13–0.37)
Female	67/39	37/78	1.00	<0.001
				0.27 (0.15–0.47)
Age				
<60	62/25	26/54	1.00	<0.001
				0.18 (0.09–0.36)
60–80	96/49	59/98	1.00	<0.001
				0.30 (0.19–0.49)
>80	17/2	5/6	1.00	0.021
				0.09 (0.01–0.69)
Smoking				
Ever	56/15	25/27	1.00	<0.001
				0.22 (0.10–0.51)
Never	119/61	65/131	1.00	<0.001
				0.25 (0.16–0.39)
Drinking				
Ever	36/13	12/19	1.00	0.002
				0.21 (0.08–0.56)
Never	139/63	78/139	1.00	<0.001
				0.25 (0.17–0.38)
Diabetes				
Yes	31/11	18/20	1.00	0.049
				0.36 (0.13–0.99)
No	144/65	72/138	1.00	<0.001
				0.24 (0.16–0.36)
Family history of cancer				
Yes	38/9	17/25	1.00	<0.001
				0.11 (0.04–0.33)
No	127/67	73/133	1.00	<0.001
				0.28 (0.19–0.42)

^a^ Adjusted for gender, age, tobacco smoking, alcohol consumption, diabetes, and family history of cancer (besides stratified factors accordingly) in unconditional logistic regression.

### 3.4 Association between ABCB1 haplotypes and PDAC susceptibility

After identifying the genotypes of the three SNPs of ABCB1, eight haplotypes were constructed using PHASE version 2.1 software. Table 4 shows the distribution of the estimated haplotypes in each group. It implied that haplotypes ACC (rs4148737A‐rs1045642C‐rs3789243C) and ATC (rs4148737A‐rs1045642T‐rs3789243C) were present more frequently in patients with PDAC compared with the control group (18.11% versus 12.87%; 33.94% versus 22.09%, respectively), whereas haplotypes GCC (rs4148737G‐rs1045642C‐rs3789243C) and GTC (rs4148737G‐rs1045642T‐rs3789243C) were present less frequently in the patients with PDAC compared with the control group (11.93% versus 19.27%; 0.64% versus 12.68%, respectively). Using unconditional logistic regression models adjusted for the confounding factors, the association between haplotypes and PDAC risk was calculated and is shown in Table 5. Carriers with haplotype ACC (a/‐ + a/a) or ATC (c/‐ + c/c) were observed to have increased susceptibility to PDAC (OR = 1.58, 95% CI: 1.02–2.45; OR = 2.16, 95% CI: 1.47–3.18, respectively), but patients with haplotype GCC (e/‐ + e/e) or GTC (g/‐ + g/g) had reduced susceptibility (OR = 0.46, 95% CI: 0.30–0.71; OR = 0.04, 95% CI: 0.01–0.14, respectively). None of the other haplotypes presented any significant differences (*p* > .05).

**TABLE 4:** Distribution of the estimated haplotype frequencies

Haplotypes	SNP positions			Cases (*n* = 228)		Controls (*n* = 217)	
	rs4148737	rs1045642	rs3789243	*n*	%^a^	*n*	%^a^
1	A	C	C	82	18.11	56	12.87
2	A	C	T	77	16.9	60	13.92
3	A	T	C	155	33.94	96	22.09
4	A	T	T	44	9.55	32	7.35
5	G	C	C	54	11.93	84	19.27
6	G	C	T	34	7.44	45	10.39
7	G	T	C	3	0.64	55	12.68
8	G	T	T	7	1.49	6	1.43

^a^ Calculated by PHASE 2.1 software.

**TABLE 5:** Association between the related haplotypes and PDAC risk

Haplotype	Cases (*n* = 228)		Controls (*n* = 217)		Crude OR (95% CI)	*p*	Adjusted OR^a^ (95% CI)	*p* ^a^
	*n*	%	*n*	%				
a = ACC								
−/−^b^	159	69.74	172	80.18	1.00	—	1.00	—
a/− + a/a	69	30.26	45	19.82	1.66 (1.08–2.56)	.022	**1.58 (1.02–2.45)**	**.041**
b = ACT								
−/−	164	71.93	167	79.96	1.00	—	1.00	—
b/− + b/b	64	28.07	50	23.04	1.30 (0.85–2.00)	.225	1.28 (0.83–1.97)	.271
c = ATC								
−/−	97	37.28	136	62.67	1.00	—	1.00	—
c/− + c/c	131	62.72	81	37.33	2.27 (1.56–3.32)	0	**2.16 (1.47–3.18)**	**<.001**
d = ATT								
−/−	189	82.89	191	88.02	1.00	—	1.00	—
d/− + d/d	39	17.11	26	11.98	1.52 (0.89–2.59)	.128	1.42 (0.82–2.44)	.212
e = GCC								
−/−	184	80.7	140	64.52	1.00	—	1.00	—
e/− + e/e	44	19.3	77	35.48	0.43 (0.28–0.67)	0	**0.46 (0.30–0.71)**	**<.001**
f = GCT								
−/−	199	87.28	178	82.03	1.00	—	1.00	—
f/− + f/f	29	12.72	39	17.97	0.67 (0.39–1.12)	.125	0.66 (0.39–1.11)	.119
g = GTC								
−/−	225	98.68	168	77.42	1.00	—	1.00	—
g/− + g/g	3	1.32	49	22.58	0.05 (0.01–0.15)	0	**0.04 (0.01–0.14)**	**<.001**
h = GTT								
−/−	221	96.93	211	97.24	1.00	—	1.00	—
h/− + h/h	7	3.07	6	2.76	1.11 (0.37–3.37)	.849	1.01 (0.33–3.11)	.99

Bold values in this table indicates the significant differences (*P* < .05).

^a^ Unconditional logistic regression adjusted for risk factors (age, gender, tobacco smoking, alcohol consumption, diabetes, and family history of cancer).

^b^ Indicates any haplotype, for example: a/− denotes the haplotype a = ACC combined with any other haplotypes.

## 4 DISCUSSION

Between 2014 and 2018, the 5‐year survival rate of pancreatic cancer increased from 6% to 9% (Bray et al., 2018), suggesting that although progress is being made, there is still a need to improve the treatments and outcomes. Pancreatic cancer remains one of the most lethal malignancies, with poor prognoses and a mortality/incidence ratio of 94% (Bray et al., 2018). Due to its atypical clinical features, nonspecific, diagnostic tools which lack specificity and no definitive cures for patients with advanced‐stage pancreatic cancer, the 1‐year survival rate of pancreatic cancer has been significantly reduced from 25% to <5% (Sarnecka, Zagozda, & Durlik, 2016). Despite advances in our understanding of this disease and potential risk factors, and advances in diagnostic tools to improve early detection, the incidence is estimated to increase with a predicted 355,317 new cases globally in 2040 (Rawla et al., 2019). Thus, it is necessary to improve our understanding of the pathogenesis of PDAC to improve the preventative, diagnostic, and therapeutic approaches. In particular, discovering new tumor biomarkers is of great significance as these biomarkers are valuable tools for improving the accuracy of diagnosis, prevention, and therapy. Among the pathogenic factors underlying the development of cancer, SNPs have been demonstrated to be associated with altered function of methylation enzymes, folate metabolism, inflammation, cell cycle, DNA repair, and also oncogenes, which are suspected to serve a role in the carcinogenesis of PDAC.

ABCB1 is an efflux protein pump, which transports toxic endogenous substances, drugs, and xenobiotics out of normal tissues and cancer cells (Hartmann, Kim, & Piquette‐Miller, 2001). The intronic SNPs at rs4148737 map to a weakly transcribed region, but also overlaps with a weak enhancer in GM12878 and in a RUNX3 ChIP‐seq cluster in the same lymphoblastoid cell line. It was predicted to overlap with a DNase hypersensitive region in the lymphoblastoid cell line and other cell lines and alter EBF, ERα‐a, and Hic1 regulatory motifs (Ruiz‐Pinto et al., 2016). Cells with the wild‐type SNPs of *ABCB1* showed drug resistance in a pancreatic cancer cell line (Kasuya et al., 2012), and polymorphic variants of *ABCB1* were observed to predict improved therapeutic efficacy and survival for patients with potentially resectable pancreatic cancer (Tanaka, Okazaki, Suzuki, Abbruzzese, & Li, 2011). However, there are no studies on the effect of its genetic polymorphism on susceptibility to PDAC, to the best of our knowledge. In the present study, the frequencies of genotypes AG and GG, as well as allele G in *ABCB1* rs4148737 were significantly lower in patients with PDAC compared with the controls. Therefore, these variant genotypes and alleles may decrease an individual susceptibility to PDAC. This interpretation of the result agrees with ABCB1’s possible protective functions and is similar to previous studies on outcomes of treatments in patients with Ewing sarcoma (Ruiz‐Pinto et al., 2016) and osteosarcoma survival following chemotherapy (He et al., 2013). Stratification by sex, age, smoking status, drinking status, diabetes, and family history of cancer showed that individuals with genotypes AG and GG still exhibited a reduced risk of developing PDAC, highlighting the protective role of these SNPs.

Additionally, although *NAMPT* rs2505568 and rs9034 are located in the 3’ untranslated region, which may affect NAMPT expression, their polymorphisms were found to be independent of the development of dilated cardiomyopathy, esophageal squamous cell carcinoma (ESCC), and bladder cancer (Dou et al., 2015; Zhang et al., 2015; Zhang, Zhou, et al., 2014). *APOB* rs1997623 and rs12672038 were reported to have no influence on ESCC or gastric cancer (Rawla et al., 2019; Zhang, Hu, et al., 2014). As these four SNPs are not associated with PDAC, these results are consistent with previous studies.

The other SNPs tested in the present study were determined to not be significantly associated with the development of PDAC, which differs from previous studies in different types of cancer. A silent mutation at *ABCB1* rs1045642 may decrease the expression levels of the mRNA and protein products, or alter the protein structure and substrate affinity (Cascorbi, 2006; Kimchi‐Sarfaty et al., 2007). Genotype TT was found to significantly increase the overall risk of gastric cancer in Iranian individuals and upper aerodigestive tract cancer in Indian individuals (Sabahi et al., 2010; Sam et al., 2007), and was associated with colorectal cancer and multiple myeloma in French individuals (Falkowski et al., 2017; Razi, Anani Sarab, Omidkhoda, & Alizadeh, 2018). There is a correlation between SNPs at rs3789243 with colorectal cancer (Andersen et al., 2013). Furthermore, SNPs at *APOB* rs693 resulting in amino acid substitution and SNPs at rs104203, which result in a silent mutation, have been reported to be associated with dyslipidemia (Tsunoda, Harihara, Tanabe, & Dashnyam, 2012) and an increased risk of breast cancer in Chinese individuals (Liu et al., 2013). *CAV1* rs3807987 and rs7804372 are located in introns, but intron polymorphisms may influence CAV1 expression levels and protein function by compounding with nearby polymorphisms, alternative splicing, and affecting the stability of mRNA during the progression of the cancer. These two SNPs are associated with susceptibility to ESCC, breast, and gastric cancer (Wang et al., 2014; Yan et al., 2019; Zhang, Hu, et al., 2014). *NAMPT* rs61330082 in the promoter region may influence NAMPT structure, function, or expression, which is similar to its role in increasing the risk of ESCC and bladder cancer (Zhang et al., 2015; Zhang, Zhou, et al., 2014).

The varying results of the seven SNPs and their effects on tumorigenesis between the present and previous studies may primarily be due to different tumor locations, tumor pathological status, tumor differentiation status, demographics, and limited number of samples available.

To analyze the combined effects of multiple loci, haplotype analyses were used to assess genetic susceptibility with increased accuracy. Based on the genotypes at *ABCB1* rs4148737A, rs1045642, and rs3789243, the presence of haplotypes ACC and ATC was constructed and positively correlated with the development of PDAC, whereas haplotypes GCC and GTC protected carriers from PDAC. Thus, the impact of allele G or A at rs4148737 among the three SNPs was thus determined to be important, which is consistent with the positive effect of allele A on PDAC tumorigenesis.

Due to a lower incidence, atypical clinical manifestations, insensitive early diagnostic tools, rapid development of the cancer, and limited treatment regimens for patients with advanced‐staged cancer, the number of PDAC cases is significantly lower compared with other types of cancer of the digestive system, particularly cases with identified pathological results. The strengths of the present study include the relatively large sample size from the same geographical area. However, the present study has some limitations. Sample size determination was not based on power calculations, which may affect the accuracy of the results to a certain extent, particularly as the sample sizes were smaller when the patients were stratified. These results need to be further confirmed using a larger sample from patients in regions with a higher incidence of PDAC, and from different ethnicities. Additionally, if the study participants were relatively homogeneous in terms of general characteristics, genetic backgrounds, and environmental risk factors, the results would be more accurate. Gene–environment interaction studies and functional studies are required to confirm these results and determine the effects of *ABCB1* genetic polymorphisms on PDAC.

In summary, the present study showed that genetic polymorphism of *ABCB1* rs4148737 was significantly associated with PDAC susceptibility in Chinese patients. Genotypes AG and GG, allele G, as well as haplotypes GCC and GTC may be protective predictive factors for PDAC. Therefore, SNPs at rs4148737 may serve as an efficient genetic susceptibility marker, and possibly a potential target for development of tools for early diagnosis, genetic prediction, and individualized genetic therapy in the future.

## ETHICS APPROVAL AND CONSENT TO PARTICIPATE

The present study was approved by the Ethics Committee of Shandong Provincial Qianfoshan Hospital and written informed consent was obtained from all participants.

## COMPETING INTERESTS

The authors declare that they have no competing interests.

## AUTHOR CONTRIBUTION

Zhang, C. devised and supervised the project, and drafted the manuscript. Li, B. and Wang, J. were involved in carrying out the experiment. Zhang, M. performed the experimental data and statistical analysis. Liu, C. and Chen, Z. aided in interpreting the results and worked the manuscript.
